# Left atrial stiffness is superior to volume and strain parameters in predicting elevated NT-proBNP levels in systemic sclerosis patients

**DOI:** 10.1007/s10554-019-01621-w

**Published:** 2019-05-15

**Authors:** Adél Porpáczy, Ágnes Nógrádi, Vivien Vértes, Margit Tőkés-Füzesi, László Czirják, András Komócsi, Réka Faludi

**Affiliations:** 1 Heart Institute, Medical School, University of Pécs, Ifjúság u. 13., Pécs, 7624 Hungary; 2 Department of Laboratory Medicine, Medical School, University of Pécs, Ifjúság u. 13., Pécs, 7624 Hungary; 3 Department of Rheumatology and Immunology, Medical School, University of Pécs, Akác u. 1., Pécs, 7632 Hungary

**Keywords:** Systemic sclerosis, Heart failure, Filling pressure, Left atrial strain, Left atrial stiffness

## Abstract

Heart failure with preserved ejection fraction (HFpEF) is common in systemic sclerosis (SSc) and implies a worse prognosis therefore non-invasive assessment of left ventricular (LV) filling pressure is pivotal. Besides E/eʹ the use of maximal left atrial volume (LA Vmax index) is recommended. LA reservoir strain was also reported to be useful. The utility of LA stiffness, however, was never investigated in SSc. Thus we aimed to compare the diagnostic power of LA Vmax index, reservoir strain and stiffness in predicting elevated LV filling pressure in SSc patients. 72 SSc patients (age: 57 ± 11 years) were investigated. LA stiffness was calculated as ratio of E/eʹ to LA reservoir strain. Elevated LV filling pressure was defined as NT-proBNP > 220 pg/ml. Receiver-operating characteristic (ROC) curves were used to estimate the diagnostic performance of the investigated parameters. Average NT-proBNP level was 181 ± 154 pg/ml. NT-proBNP > 220 pg/ml was found in 21 SSc patients. LA stiffness showed the highest diagnostic performance in predicting NT-pro-BNP > 220 pg/ml, with a cut off value of 0.314 (Area under the curve: 0.719, specificity: 89.4%, sensitivity: 42.1%). AUC values for LA reservoir strain and Vmax index were 0.595 and 0.521, respectively. LA stiffness was superior to Vmax index and reservoir strain in predicting elevated NT-proBNP levels in SSc patients. Although invasive validation studies on larger samples are required, our data suggest, that the use of LA stiffness may significantly contribute to diagnostic precision in populations with a high suspicion of HFpEF.

## Introduction

Cardiac involvement is an important adverse finding in systemic sclerosis (SSc). Epidemiologic studies show that it is responsible for 20–30% of all premature deaths in these patients [1]. Left ventricular (LV) systolic dysfunction is not common in SSc [2]. Diastolic dysfunction and the consequential heart failure with preserved ejection fraction (HFpEF) are much more frequent as they reflect the primary myocardial involvement of the disease [3]. These factors are also proved to be associated with increased risk of mortality [4–6]. Thus assessment of LV diastolic function and filling pressure has important diagnostic and prognostic implications in SSc. In addition to the invasive measurements, N-terminal pro-brain natriuretic peptide (NT-proBNP) levels provide reliable estimation of elevated LV filling pressure [7–9]. In the everyday practice, however, echocardiography is used for this purpose. The E/eʹ ratio is the most thoroughly studied index characterizing LV filling pressure and is included into the algorithms of all the relevant authoritative documents [10–12]. Nevertheless, recent studies have challenged the accuracy of E/eʹ in patients with or at risk for HFpEF [13–17]. Thus additional echocardiographic parameters are also required for identifying elevated LV filling pressure. The current recommendations suggest the use of the maximal left atrial (LA) volume index [12], as it is a reliable indicator of the duration and severity of the elevated filling pressure [18]. Recent studies proved, however, that 2-dimensional speckle tracking-derived LA reservoir strain also shows a good correlation with LV filling pressure [19–21], exceeding the diagnostic power of the maximal LA volume [22, 23]. LA stiffness is a further parameter of the atrial performance, representing the change in pressure required to increase the volume of the atrium in a given measure [24, 25]. It was reported as a useful index to distinguish HFpEF patients from those with asymptomatic diastolic dysfunction [25].

Thus we aimed to compare the diagnostic power of the maximal LA volume index, LA reservoir strain and LA stiffness in predicting elevated LV filling pressure in SSc patients. NT-proBNP served as non-invasive measure of the LV filling pressure in our study.

## Methods

### Study population

Our prospective study included 80 consecutive SSc patients diagnosed in the tertiary centre of the Department of Rheumatology and Immunology, University of Pécs. All enrolled patients needed to comply the American College of Rheumatology criteria for SSc and were categorized as suffering from limited cutaneous or diffuse cutaneous SSc according to the criteria defined by LeRoy et al. [26]. All patients fulfilled the recently revised ACR/EULAR classification criteria [27]. We performed extensive assessment of the medical history. Subjects with atrial fibrillation, pulmonary arterial hypertension, known coronary artery disease, cardiomyopathies or significant left sided valvular disease were excluded from our study. Time between the beginning of the first non-Raynaud symptom of SSc and the echocardiographic analysis was defined as duration of the disease. Limitations of physical activity were graded according to the New York Heart Association classification. 6-minute walk test was carried out on the same day as the echocardiographic measurements. All work was done in compliance with the Declaration of Helsinki and was performed with the approval of the institutional ethics committee. Written informed consent was obtained from all patients.

### NT-proBNP measurements

Blood samples were obtained immediately prior to the echocardiographic studies. Plasma concentrations of NT-proBNP were analysed by electrochemiluminescence immunoassay (*Elecsys 2010 system,Roche Diagnostics, Mannheim, Germany*). NT-proBNP value > 220 pg/ml was defined as the evidence of the elevated LV filling pressure [10].

### Echocardiography

All patients underwent echocardiographic examination performed by a single investigator using Philips EPIQ 7 ultrasound system (Philips Healthcare, Best, The Netherlands). LV ejection fraction was calculated by biplane Simpson’s method. In addition to the LV end-diastolic diameter, end-diastolic thickness of the septum and posterior wall were measured from parasternal long axis view, using M-mode. LV mass was calculated based on the Devereux formula and then indexed for body surface area (LVM index) [28]. Mitral regurgitation was evaluated according to the recent guidelines and categorized as mild, moderate, or severe [29]. Transmitral flow velocities (E, A) as well as myocardial systolic (S), early-(eʹ) and late-(aʹ) diastolic velocities were measured at the lateral and septal border of the mitral annulus. Lateral and septal myocardial velocities were averaged. Mitral E/A and E/eʹ ratios were computed. E/eʹ > 14 was regarded as elevated, while values between 10 and 14 were considered as “grey zone” values [12]. Systolic pulmonary artery pressure (PASP) was calculated from tricuspid regurgitation velocity added to the right atrial pressure (5 to 15 mmHg) estimated using the diameter and collapsibility index of the inferior vena cava [28]. Doppler measurements were acquired from ≥ 3 consecutive heart cycles.

### Strain measurements

**Fig. 1:**
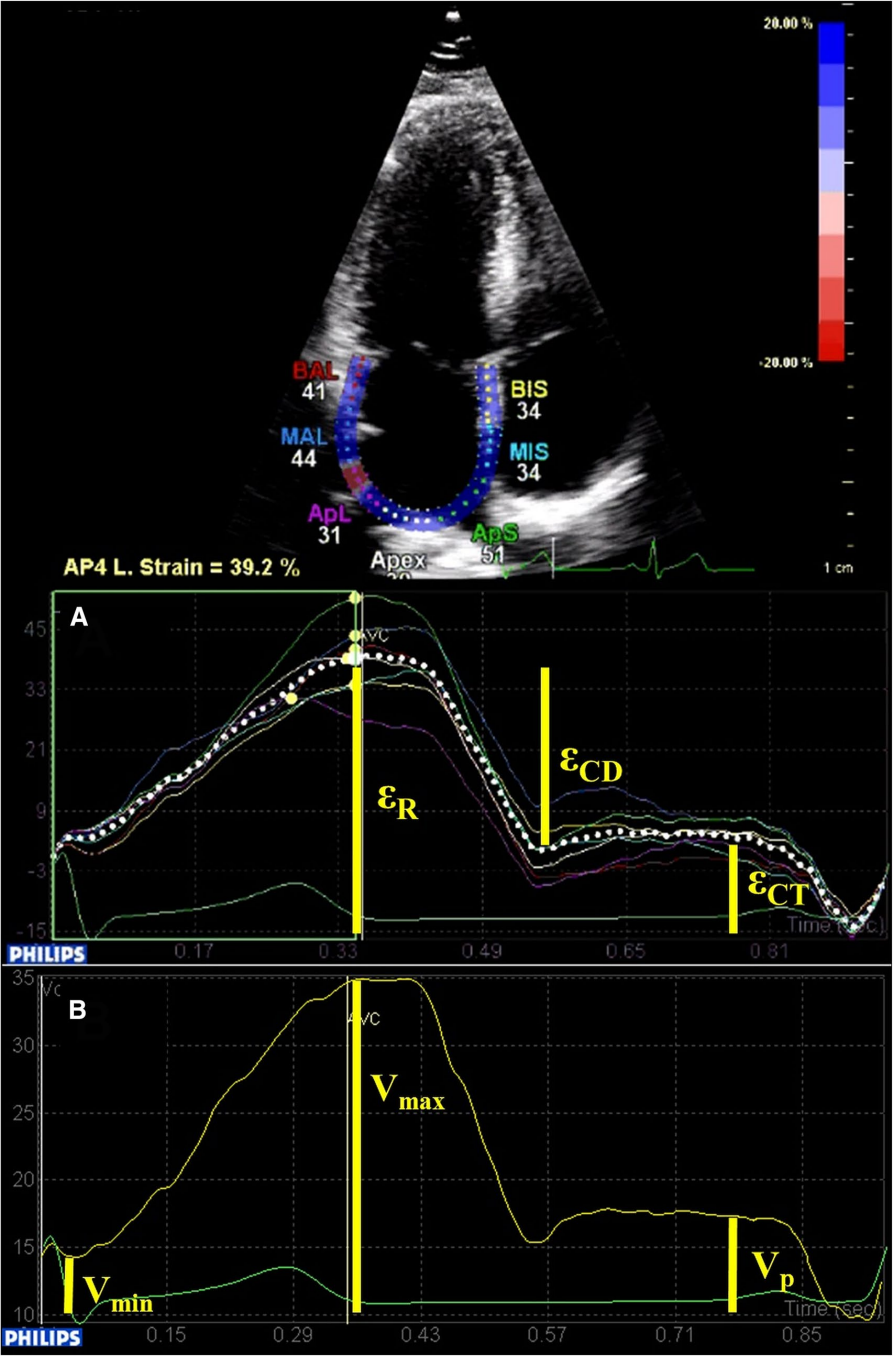
Four-chamber view image depicting the analysis of LA strain using speckle tracking technique. The region of interest is optimized manually, and then LA strain curve is created by the speckle-tracking software (**a**). Using the atrial borders created for speckle tracking analysis, LA volume curves are generated by the same software (**b**). *ε*_*R*_ Reservoir strain, *ε*_*CD*_ conduit strain, *ε*_*CT*_ contractile strain, *Vmax* maximal volume, *Vmin* minimal volume, *Vp* volume at the beginning of P wave)

LA-focused two-dimensional echocardiographic images were obtained from apical four-, and two-chamber views for speckle tracking analysis. Care was taken to obtain true apical images and to avoid foreshortening. The frame rate was set between 80 and 90 frames/s to ensure adequate speckle-tracking. For each view three cardiac cycles were recorded and stored. A dedicated software (QLab, Philips Healthcare, Andover, MA, USA) was used for offline analysis by a single investigator, blinded to the echocardiographic and clinical data. In segments with insufficient tracking, manual readjustment of the endocardial border was applied to optimize tracking quality. The onset of R-wave on the electrocardiographic trace was used as zero-reference point of the strain analysis. LA reservoir strain was defined as the peak systolic strain, just before mitral valve opening. This was followed by a plateau and a second late peak at the onset of the P-wave indicating the contractile strain. Conduit strain was calculated as the difference between reservoir and contractile strain **(**Fig. 1a**)**. Data obtained in the two views were averaged [30]. LA stiffness was calculated as ratio of E/eʹ to LA reservoir strain [24, 25].

### Volumetric measurements

LA volume curves were generated by the same software using the endocardial borders created for speckle tracking analysis. The following LA volumes were obtained: maximal LA volume (Vmax) at the end of the T-wave on the electrocardiogram, just before the mitral valve opening; minimal LA volume (Vmin) at the QRS complex, just after the mitral valve closure; and volume at atrial contraction (Vp) at the beginning of P-wave **(**Fig. 1b**).** Values from the two views were averaged and indexed for body surface area (Vmax-, Vmin- and Vp index) [31].

### Statistical analysis

Categorical data were expressed as frequencies and percentages; continuous data were expressed as the mean ± SD. Comparisons of data between two groups were performed using independent-sample t-tests or independent Mann–Whitney test for continuous variables and chi square tests for categorical variables.

Since concentration of NT-proBNP did not show normal distribution, logarithmic transformation was performed. Relationship between lnNTproBNP and the investigated echocardiographic parameters was assessed using linear regression analysis. Potential determinants of the NT-proBNP level (age, body surface area, estimated glomerular filtration rate, LV ejection fraction, and duration of the disease) were also included into the analysis. In the second step, multiple stepwise linear regression analysis was performed, by entering those variables with p < 0.1 in the univariate analysis. Variance Inflation Factor (VIF) values above 2.5 were considered to have potential multicollinearity.

Receiver-operating characteristic (ROC) curves were used to examine the diagnostic performance of the echocardiographic parameters in predicting elevated LV filling pressure. Area under the curve (AUC) values were calculated. Sensitivity and specificity were computed for LA stiffness using various possible cut-off points.

Intraobserver and interobserver variability was assessed by the intraclass correlation coefficient. A p value of < 0.05 was considered significant. Data were analysed using IBM SPSS 22 statistical software.

## Results

From the total cohort of 80 participants, 72 were eligible for the study. Eight patients were excluded due to LA foreshortening (n = 3), or inadequate acoustic window (n = 5). Intraclass correlation coefficients for intraobserver variability were 0.982, 0.945, 0.908, 0.944, 0.903, and 0.913 for reservoir, conduit and contractile strain, and Vmax, Vp, and Vmin, respectively. Regarding interobserver variability, intraclass correlation coefficients for reservoir, conduit and contractile strain, and Vmax, Vp, and Vmin were 0.974, 0.932, 0.898, 0.931, 0.899 and 0.882, respectively. The mean age was 57.1 ± 11.3 years, 66 (92%) were female. LV ejection fraction was preserved (≥ 55%) in 70 (97%) and mildly reduced (45–54%) in 2 (3%) patients. Detailed clinical and echocardiographic data of the 72 patients are reported in Table 1. NT-proBNP > 220 pg/ml was found in 21 (29%) patients. Characteristics of our study cohort stratified by this NT-proBNP level are shown in Table 1.

**Table 1:** Characteristics of the study population

	All patients (n = 72)	NT-proBNP ≤ 220 pg/ml (n = 51)	NT-proBNP > 220 pg/ml (n = 21)	p
Clinical characteristics				
Age (year)	57.1 ± 11.3	54.5 ± 11.7	63.2 ± 7.3	** < 0.001**
Female gender n (%)	66 (92)	46 (90)	20 (95)	0.482
Body mass index (kg/m^2^)	25.9 ± 5.0	26.3 ± 4.7	25 ± 5.7	0.328
Body surface area (m^2^)	1.7 ± 0.2	1.7 ± 0.2	1.7 ± 0.2	0.933
DcSSc (%)	39 (54)	25 (49)	14 (67)	0.172
Duration of the disease (year)	7.3 ± 5.9	6.3 ± 5.3	9.7 ± 6.8	**0.031**
NYHA class				0.080
Class I n (%)	22 (31)	17 (33)	5 (24)	
Class II n (%)	32 (44)	25 (49)	7 (33)	
Class III n (%)	18 (25)	9 (18)	9 (43)	
6MWT distance (m)	396 ± 94	410 ± 96	360 ± 83	**0.041**
NT-proBNP (pg/ml)	181.4 ± 153.9	97.6 ± 44.7	384.7 ± 133.2	** < 0.001**
Comorbidities				
Systemic arterial hypertension n (%)	33 (46)	23 (45)	10 (48)	0.849
eGFR (ml/min/1.73m^2^)	87.3 ± 24.6	94.4 ± 21.6	70.1 ± 23.0	** < 0.001**
Echocardiographic characteristics				
LV ejection fraction (%)	60.1 ± 4.6	61.6 ± 3.4	59.1 ± 5.5	**0.039**
LVM index (g/m^2^)	97.0 ± 19.5	95.8 ± 21.3	99.7 ± 14.4	0.370
Grade of mitral regurgitation				**0.035**
Mild (n) %	66 (92)	49 (96)	17 (81)	
Moderate (n) %	6 (8)	2 (4)	4 (19)	
Severe (n) %	0 (0)	0 (0)	0 (0)	
PASP (mmHg)	26.7 ± 7.5	25.3 ± 5.7	29.6 ± 10.1	0.062
Mitral E (cm/s)	73.8 ± 18.0	72.0 ± 16.5	78.3 ± 21.1	0.187
Mitral A (cm/s)	72.4 ± 20.4	67.9 ± 17.6	84.1 ± 22.5	**0.002**
Mitral E/A	1.1 ± 0.4	1.1 ± 0.4	0.95 ± 0.2	**0.020**
Averaged mitral annular S (cm/s)	8.3 ± 1.3	8.4 ± 1.2	8.0 ± 1.5	0.218
Averaged mitral annular eʹ (cm/s)	8.3 ± 2.0	8.6 ± 2.1	7.5 ± 1.6	**0.040**
Averaged mitral annular aʹ (cm/s)	9.8 ± 1.6	9.9 ± 1.6	9.5 ± 1.6	0.295
Mitral E/eʹ	9.4 ± 2.8	8.7 ± 2.3	11 ± 3.4	**0.001**
LA parameters				
Vmax index (mL/m^2^)	25 ± 7.7	24.7 ± 7.8	25.6 ± 7.7	0.672
Vmin index (mL/m^2^)	11.8 ± 5.2	11.5 ± 4.7	12.4 ± 6.3	0.474
Vp index (mL/m^2^)	16.2 ± 6.6	16.0 ± 6.3	16.7 ± 7.3	0.701
Reservoir strain (%)	41.1 ± 8.2	41.9 ± 8.1	39 ± 8.2	0.178
Conduit strain (%)	22.3 ± 6.5	22.8 ± 6.7	20.9 ± 5.8	0.218
Contractile strain (%)	18.8 ± 4.1	19.1 ± 4.2	18.1 ± 3.9	0.372
Stiffness	0.245 ± 0.12	0.219 ± 0.08	0.311 ± 0.16	**0.024**

Statistically significant p-values are formatted in bold (p < 0.05)

*DcSSc* Diffuse cutaneous form of systemic sclerosis, *NYHA* New York Heart Association, *6MWT* six-minute walk test, *eGFR* estimated glomerular filtration rate, *LV* left ventricular, *LVM* left ventricular mass, *PASP* pulmonary artery systolic pressure, *LA* left atrial

Patients with elevated NT-proBNP levels were significantly older and their walking distance was significantly shorter compared with the other subgroup. The course of the SSc was significantly longer in this population. The difference in LV ejection fraction was clinically not remarkable. Significantly higher E/eʹ values were found in the patients with elevated NT-proBNP levels: E/eʹ > 14 was found in 5 (24%) patients, while in 10 (48%) patients E/eʹ values were in the “grey zone” (between 10 and 14) in this subgroup. LA Vmax index and reservoir strain values were similar in the two subgroups. LA stiffness, on the other hand, was significantly elevated in the subgroup of patients with high NT-proBNP values.

Univariate and multivariate predictors of the NT-proBNP level are reported in Table 2. In stepwise multiple linear regression analysis estimated glomerular filtration rate, LA stiffness and LV ejection fraction became independent predictors of the NT-proBNP level (multiple r = 0.614; p = 0.000; F = 13.537). VIF values for all variables were below 2.5.

**Table 2:** Predictors of the (ln) NT-proBNP in univariate and multivariate regression analyses

	Univariate analysis		Multivariate analysis	
	r	p	β	p
Age (years)	0.384	**0.001**		
Body surface area (m^2^)	− 0.160	0.178		
Duration of the disease (y)	0.233	**0.049**		
eGFR (ml/min/1.73 m^2^)	− 0.502	** < 0.001**	− 0.409	** < 0.001**
LV ejection fraction (%)	− 0.209	0.079	− 0.194	**0.048**
LA Vmax index (mL/m2)	0.285	**0.015**		
LA reservoir strain (%)	− 0.238	**0.044**		
LA Stiffness	0.431	** < 0.001**	0.287	**0.007**

Statistically significant p-values are formatted in bold (p < 0.05)

*eGFR* estimated glomerular filtration rate, *LV* left ventricular, *LA* left atrial

**Fig. 2:**
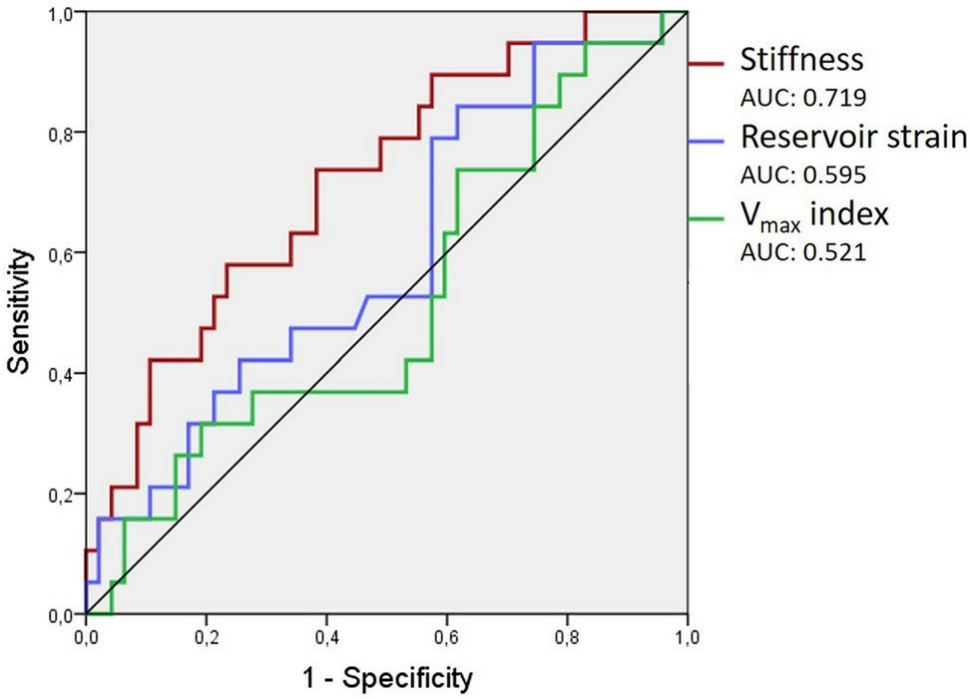
ROC curves for maximal LA volume index, LA reservoir strain and LA stiffness for the prediction of NT-proBNP > 220 pg/ml

Using ROC analysis, LA stiffness showed the highest diagnostic performance in predicting NT-pro-BNP > 220 pg/ml, with an AUC of 0.719. ROC curves demonstrating the predictive power of the three LA parameters are presented in Fig. 2.

**Fig. 3:**
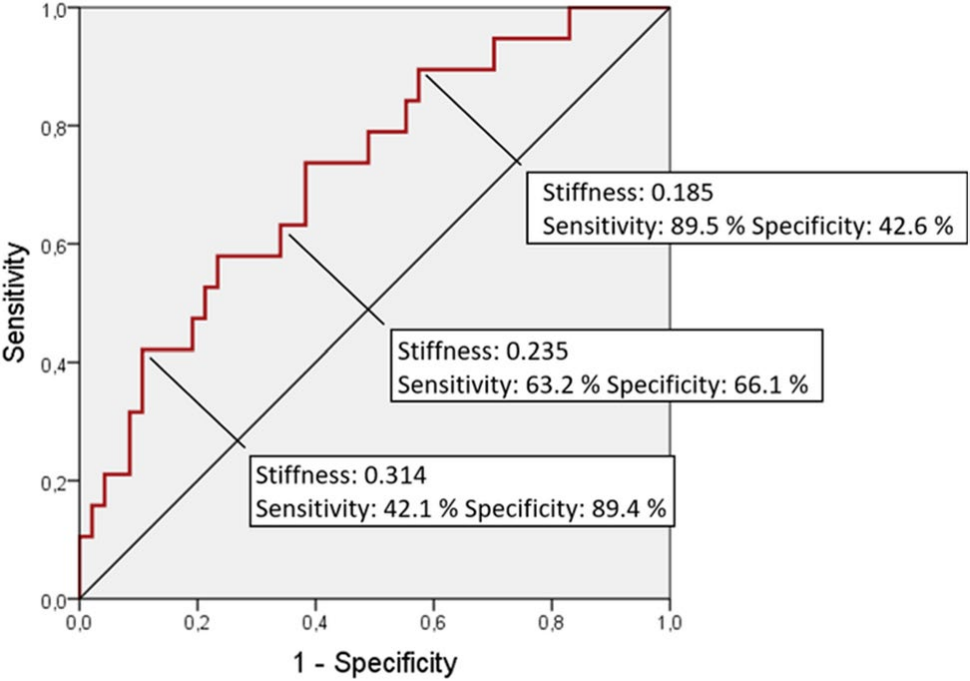
ROC curve displaying the sensitivity and specificity of various LA stiffness values in predicting NT-proBNP > 220 pg/ml

Sensitivity and specificity values were computed for LA stiffness using various possible cut-off points **(**Fig. 3**)**. LA stiffness with the cutoff value of 0.314 showed a high specificity (89.4%) in predicting NT-pro-BNP > 220 pg/ml, with a sensitivity of 42.1%.

## Discussion

The main finding of our study is that LA stiffness is superior to volume and strain parameters in predicting elevated NT-proBNP levels in patients with SSc.

Overt LV systolic dysfunction is rare in SSc [2]. Still, heart failure is a typical manifestation of the cardiac involvement in this disease. Primary myocardial involvement is thought to be the consequence of the repeated focal ischemic injuries resulting in irreversible myocardial fibrosis [3, 32]. If myocardial fibrosis progresses, diastolic compliance of the LV decreases and manifest HFpEF may evolve. Numerous characteristic symptoms of SSc patients (impaired functional capacity, dyspnoea, peripheral oedema) are definitely related to LV diastolic dysfunction and elevated filling pressure. In addition, these factors are proved to be associated with increased risk of mortality [4–6]. Heart failure symptoms in SSc, however, may be misinterpreted as pulmonary arterial hypertension or interstitial lung disease, leaving HFpEF underdiagnosed. A diagnostic hallmark of heart failure is elevated LV filling pressure, a compensatory response to sustain cardiac output. Thus assessment of LV filling pressure has important diagnostic and prognostic implications in this disease. Although cardiac catheterization remains the gold standard and elevated NT-proBNP levels may also be useful, echocardiography is usually the first test to perform. Thus there is a continuing search for non-invasive markers of elevated LV filling pressure. The previously used parameters have several limitations and reflect different physiological aspects of the diastole. E/eʹ—the ratio of the early diastolic velocity of the mitral inflow to early diastolic velocity of the mitral annulus—provides a close approximation of LV filling pressures in a wide spectrum of diseases and its prognostic value has also been proved. Nevertheless, strength of correlation between E/eʹ and LV filling pressure varied widely between studies [13–17]. Particularly weak correlations were observed in the so called grey zone (average E/eʹ between 10 and 14 [12]; septal E/eʹ between 8 and 15 [33]; lateral E/eʹ between 8 and 12 [34]). Thus additional echocardiographic parameters are also required for identifying elevated LV filling pressure. When indexed to body surface area, maximal LA volume has been proposed as a biomarker of the severity and duration of the elevated filling pressure, especially in patients without significant valvular heart disease or history of atrial fibrillation [18]. It has also been reported as an independent predictor of the cardiovascular outcome in the general population [35] and in SSc [5]. Increased LA volume is also known as an independent predictor of raised NT-proBNP levels in HFpEF patients [36]. Thus the current recommendation of the American Society of Echocardiography and the European Association of Cardiovascular Imaging suggests the use of maximal LA volume index as additional parameter for the evaluation of LV filling pressure [12].

Recent studies proved, however, that the enlargement of the cavity is preceded by the functional remodelling of the LA [37–39]. Two-dimensional speckle tracking-derived LA reservoir strain showed significant correlation with the amount of LA wall fibrosis as assessed by cardiac magnetic resonance imaging and with LA interstitial fibrosis in patients with mitral valve disease in histopathologic specimens [40, 41]. This parameter showed a good correlation with the invasively measured LV filling pressure and with NT-proBNP levels, exceeding the diagnostic power of the maximal LA volume [20–23, 38]. It was also proved to be superior to LA volume as predictor of the cardiovascular events [42]. By the help of the same technique it has been reported by our group that impaired LA mechanics was an early sign of myocardial involvement in SSc, strongly reflecting the changes in LV diastolic function [37].

In our recent study, beside LA reservoir strain, we applied a further parameter of the atrial performance, LA stiffness, which has never been investigated in SSc before. This parameter is obtained by tissue Doppler and speckle tracking techniques and represents the change in pressure required to increase the volume of the atrium in a given measure [24, 25]. Kurt et al. reported LA stiffness as a useful index to differentiate between HFpEF and asymptomatic diastolic dysfunction [25]. In the study of Pilichowska-Paszkiet et al. LA fibrosis was detected by electroanatomical mapping in patients with atrial fibrillation. LA stiffness showed more robust correlation with the extent of LA fibrosis compared with LA strain [43].

Thus in our study we aimed to compare the diagnostic power of the maximal LA volume, LA reservoir strain and LA stiffness in predicting elevated LV filling pressure in SSc patients. The cut-off value of NT-proBNP > 220 pg/ml is considered to have a high positive predictive value for the diagnosis of HFpEF [10], therefore NT-proBNP served as non-invasive measure of the LV filling pressure.

Our data show that LA stiffness has higher discriminative strength in identifying patients with elevated NT-proBNP levels compared with maximal LA volume index and LA reservoir strain. Two parameters, both reflecting LV filling pressure but obtained by completely different approaches, are combined in LA stiffness. This may explain the diagnostic efficacy of this parameter. The common principle of the previous and current echocardiographic recommendations is that cut-off values with high specificity are used to avoid false positive diagnoses of diastolic dysfunction and elevated filling pressure [12, 44]. Thus we suggest the use LA stiffness with the cut-off value of 0.314 as this value showed high specificity (with modest sensitivity) in predicting elevated LV filling pressures.

Although invasive validation studies on larger samples are required, our data suggest, that LA stiffness is superior to maximal LA volume index and LA reservoir strain and may be used as one of the reliable echocardiographic parameters in recognizing SSc patients with elevated LV filling pressure.

## Limitations

Numerous limitations of our study need to be acknowledged. For obtaining LA strain values, we used a software that was developed for LV strain analysis because a dedicated software for atrial strain estimation was not available. Besides, larger sample size and prospective follow-up are needed to assess the prognostic impact of the elevated LA stiffness in SSc population. The major limitation of our study was that instead of measuring LV filling pressure invasively, it was estimated using NT-proBNP, which is known to have limited applicability in this context.

## Conclusion

LA stiffness was superior to maximal LA volume index and LA reservoir strain in predicting elevated NT-proBNP levels in our SSc patients. Although invasive validation studies on larger samples are required, our data suggest, that the use of LA stiffness may significantly contribute to diagnostic precision in populations with a high suspicion of HFpEF.
